# Effect of* Aurantii Fructus Immaturus Flavonoid* on the Contraction of Isolated Gastric Smooth Muscle Strips in Rats

**DOI:** 10.1155/2016/5616905

**Published:** 2016-06-27

**Authors:** Zhenyu Wu, Shengsheng Zhang, Peicai Li, Xiaofang Lu, Jiajia Wang, Luqing Zhao, Yueqi Wang

**Affiliations:** Digestive Disease Center, Beijing Hospital of Traditional Chinese Medicine Affiliated to Capital Medical University, Beijing 100010, China

## Abstract

This study was designed to investigate the effect of* Aurantii fructus immaturus flavonoid* (AFIF) on the contraction of isolated gastric smooth muscle in rats and explore its underlying mechanisms. Isolated antral longitudinal smooth muscle strip (ALSMS) and pyloric circular smooth muscle strip (PCSMS) of rats were suspended in tissue chambers. The responses of ALSMS and PCSMS to administration of AFIF were observed. Cyclic guanosine monophosphate (cGMP) and protein kinase G (PKG) levels of PCSMS were measured by ELISA kits. In this study, AFIF showed no significant effect on ALSMS contraction, but it dose-dependently reduced the mean contraction amplitude of PCSMS. When the concentration of AFIF reached 3000 *μ*g/mL, 6000 *μ*g/mL, and 10000 *μ*g/mL, its inhibitory effect on PCSMS contraction was significant. This effect of AFIF was weakened in Ca^2+^-rich environment. And N*ω*-nitro-L-arginine methyl (L-NAME), the inhibitor of nitric oxide synthase (NOS), significantly inhibited AFIF's action in comparison with control (*P* < 0.05). After incubation with AFIF for 30 min, levels of cGMP and PKG in PCSMS were significantly increased compared with control (*P* < 0.05). Our results suggest that AFIF has a dose-dependent diastolic effect on PCSMS in rats, which may be related to the regulatory pathway of NO/cGMP/PKG/Ca^2+^.

## 1. Introduction

Functional dyspepsia (FD) is a disease defined as persistent or recurrent postprandial upper abdominal discomfort and epigastric pain in the absence of any organic, systemic, or metabolic diseases in the Rome III criteria [1]. According to a systemic review, FD has a high prevalence of 11.5%~14.5% all over the world [2], and it markedly reduces patients' quality of life [3, 4]. Gastric emptying dysfunction is recognized as one of the pathogenic factors of FD and causes the main clinical symptoms of this disease [5]. Unfortunately, existing prokinetic drugs are still not satisfactory to promote the symptoms of FD.

Recently, therapeutic effects of Chinese herbals and their extracts on FD have been paid a close attention [6].* Aurantii fructus immaturus* (AFI), the dry fruit of* Citrus aurantium* L. and* Citrus sinensis* Osbeck, is one of the main Chinese herbals to treat gastrointestinal disorders. Previous study has shown that AFI contributes to the treatment of FD [7]. As the major effective constituent of AFI [8],* Aurantii fructus immaturus flavonoid* (AFIF) can promote gastric empting process in FD rats [9]. However, the mechanism of the therapeutic effect of AFIF is not yet clear, and AFIF's action on isolated gastric muscle strips has not been reported.

As we know, antrum and pylorus are two important functional units in gastric emptying process. The normal conduct of gastric emptying process depends on the contraction of antral muscle, the relaxing of pyloric muscle, and the coordination of these two functional units. And the pathway of NO/cGMP/PKG contributes to the regulation of gastric muscle contraction by regulating Ca^2+^ influx process.

In this paper, we studied AFIF's action on isolated antral muscle strips and pyloric muscle strips in rats to investigate whether it promotes antral muscle contraction or weakens pyloric muscle contraction. To explore the connection between AFIF's action and the influx of extracellular Ca^2+^, we studied AFIF's action in different Ca^2+^ environment. To explore the mechanisms of AFIF's action based on NO/cGMP/PKG pathway, we studied the effect of L-NAME on AFIF's action and measured the cGMP and PKG levels of muscle strips after AFIF incubation.

## 2. Materials

### 2.1. Animals

Male Sprague-Dawley rats, obtained from the Vital River Laboratories, China, and weighing 290–310 g, were used. The animals were accommodated in suitable conditions (at 22°C and fed ad libitum). The study was conducted in accordance with the principles of the National Institute of Health Guide for the Care and Use of Laboratory Animals, and permission was received from the local ethics committee of Beijing Hospital of Traditional Chinese Medicine.

### 2.2. Drugs and Reagents

AFIF (batch number: 20140305, made and identified by Jiangxi Qingfeng Pharmaceutical Co., Ltd. for Drug Control) was dispensed into different concentrations with Krebs solution, respectively. The following reagents were used: acetylcholine chloride (batch number: A6625, Sigma-Aldrich Co. LLC), neostigmine bromide (batch number: N2001, Sigma-Aldrich Co. LLC), atropine sulfate (batch number: 1306281, Beijing Hospital of Traditional Chinese Medicine), and N*ω*-nitro-L-arginine methyl (L-NAME, batch number: N5751, Sigma-Aldrich Co. LLC). Krebs solution had the following composition in mmol/L: NaCl 120.6, KCl 5.9, NaH_2_PO_4_ 1.2, MgCl_2_ 1.2, NaHCO_3_ 15.4, CaCl_2_ 2.5, and C_6_H_12_O_6_ 11.5. Rat cyclic guanosine monophosphate (cGMP) ELISA kit (batch number: KGE003, R&D systems) and rat protein kinase G (PKG) ELISA kit (batch number: EY-(Ela)-2763, Shanghai Yi Yan Biological Technology Co., Ltd., China) were used to measure the levels of cGMP and PKG.

### 2.3. Apparatus

CH-1015 super thermostatic bath was from Shanghai Yueping Scientific Instrument Co., Ltd., China. Flowing apparatus was from AD Instruments, Australia: MLT 02021D external isometric force transducer, PL 3508-0028 four-channel recorder, ML 0146/C-V Panlab Four-Chamber Organ Bath, ML110 Powerlab amplifier, and PowerLab/4SP data processing and analysis system.

## 3. Methods

### 3.1. Tissue Preparation

All rats were fasted with free access to water for 20 hours. Each rat was hit to lose consciousness and the whole stomach was removed. The stomach was opened along greater curvature and rinsed with Krebs solution. Muscle strips (8 mm × 2 mm) cut parallel to longitudinal fibers of the antrum were named as antral longitudinal smooth muscle strips (ALSMS), and muscle strips (8 mm × 2 mm) cut parallel to circular fibers of the pylorus were named as pyloric circular smooth muscle strips (PCSMS) [10]. Each strip with the mucosa removed was suspended in a tissue chamber containing 15 mL Krebs solution, constantly warmed by circulating water jacked at 37°C and supplied with a mixed gas of 95% O_2_ and 5% CO_2_. One end of each strip was fixed to a hook on the bottom of the chamber while the other end was connected by a thread to an external isometric force transducer (MLT 02021D) at the top. Each strip was subjected to 1 g load tension and washed with 15 mL Krebs solution every 15 minutes. The contractions of strips were simultaneously recorded by PL 3508-0028 four-channel recorder and analyzed by PowerLab/4SP data processing and analysis system. After equilibrating for 1 h to get a stable baseline, whose mean contraction amplitude was recognized as 100%, 5 *μ*L of normal saline (NS) was added as control (AFIF concentration was 0 *μ*g/mL). Tissue preparation was done before each experiment.

### 3.2. Experiments

#### 3.2.1. AFIF's Effect on the Contraction of Gastric Smooth Muscle Strips

AFIF was added to the bath continuously at intervals of 5 min, so that the cumulative concentration of AFIF in each chamber reached 3 *μ*g/mL, 30 *μ*g/mL, 300 *μ*g/mL, 3000 *μ*g/mL, 6000 *μ*g/mL, and 10000 *μ*g/mL step by step; the contraction amplitude and contractile frequency of each strip were observed.

#### 3.2.2. Effect of Extracellular Ca^2+^ Concentration on AFIF's Action

15 mL of Ca^2+^-lack Krebs solution (Ca^2+^ concentration was 1.25 mmol/L) or Ca^2+^-rich Krebs solution (Ca^2+^ concentration was 5 mmol/L) was put into each chamber instead of normal Krebs solution (Ca^2+^ concentration was 2.5 mmol/L). Then AFIF was added to each bath continuously at intervals of 5 min, so that the cumulative concentration of AFIF in each chamber reached 3 *μ*g/mL, 30 *μ*g/mL, 300 *μ*g/mL, 3000 *μ*g/mL, 6000 *μ*g/mL, and 10000 *μ*g/mL. The PCSMS contraction amplitude and contractile frequency were observed.

#### 3.2.3. AFIF's Action on Abnormal Contraction of Gastric Muscle Strips

Acetylcholine chloride (0.35 *μ*mol/L), neostigmine (1 *μ*mol/L), or atropine (1 *μ*mol/L) was added to the bath, respectively, to induce the abnormal contraction of PCSMS before AFIF administration. Then AFIF was added to each bath continuously at intervals of 5 min, so that the cumulative concentration of AFIF in each chamber reached 3 *μ*g/mL, 30 *μ*g/mL, 300 *μ*g/mL, 3000 *μ*g/mL, 6000 *μ*g/mL, and 10000 *μ*g/mL. The PCSMS contraction amplitude and contractile frequency were observed.

#### 3.2.4. Effect of L-NAME on AFIF's Action

Strips were incubated with L-NAME (10^−4^ mol/L) for 20 min before AFIF administration. Then AFIF was added to each bath continuously at intervals of 5 min, so that the cumulative concentration of AFIF in each chamber reached 3000 *μ*g/mL, 6000 *μ*g/mL, and 10000 *μ*g/mL. The contraction amplitude of each PCSMS was observed.

#### 3.2.5. AFIF's Effect on cGMP and PKG Levels of Gastric Muscle Strips

AFIF (6000 *μ*g/mL) or NS was added to each chamber containing PCSMS after the incubation for 30 min. Then cGMP and PKG levels of these PCSMS were measured. The testing procedures were carried out according to the instructions of ELISA kits.

### 3.3. Statistical Analysis

All experimental data were analyzed with one-way analysis of variance or independent *t*-test using SPSS 20.0 software (SPSS, Chicago, IL, USA) and expressed as mean ± SD. *P* < 0.05 was considered statistically significant.

## 4. Results

### 4.1. AFIF Showed No Significant Effect on the Contraction of ALSMS

**Figure 1:**
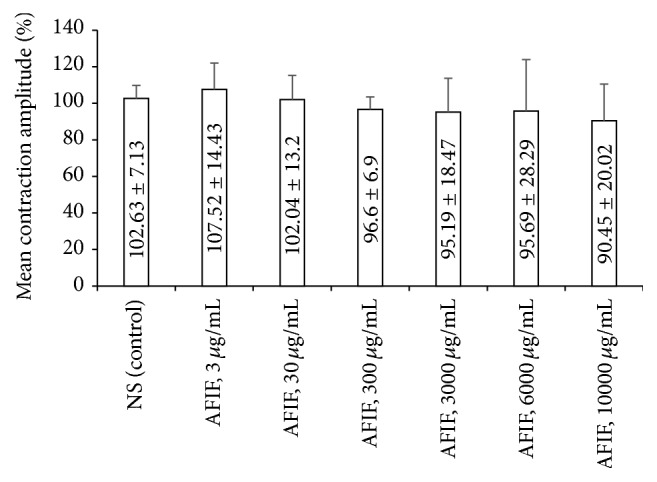
AFIF's action on the mean contraction amplitude of ALSMS. *n* = 9. The administration of AFIF (3 *μ*g/mL, 30 *μ*g/mL, 300 *μ*g/mL, 3000 *μ*g/mL, 6000 *μ*g/mL, and 10000 *μ*g/mL) did not change the mean contraction amplitude of ALSMS significantly compared with control.

**Figure 2:**
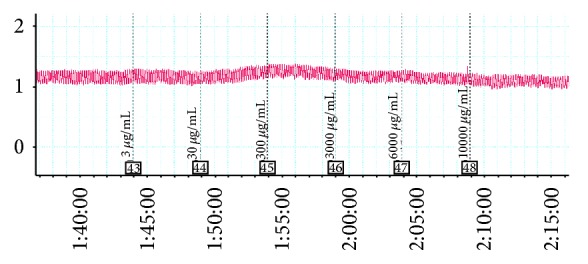
Effect of AFIF on spontaneous contraction of ALSMS recorded by PL 3508-0028 four-channel recorder. AFIF showed no significant influence on ALSMS contraction. Neither the mean contraction amplitude nor the mean contractile frequency of ALSMS changed significantly.

Administration of AFIF affects neither the mean contraction amplitude (Figures 1 and 2) nor the mean contractile frequency (Table 1 and Figure 2) of ALSMS.

### 4.2. AFIF Had a Concentration-Dependent Inhibitory Action on the Mean Contraction Amplitude of PCSMS, Which Was Negatively Correlated with Ca^2+^ Concentration

**Figure 3:**
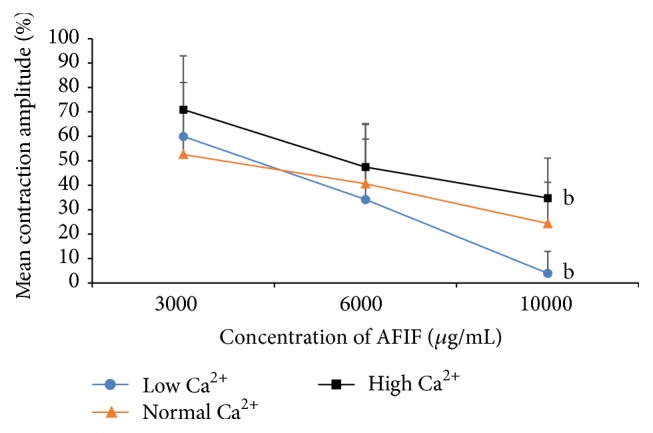
AFIF's action on PCSMS contraction amplitude is negatively correlated with Ca^2+^ concentration. *n* = 10, ^b^ *P* < 0.01 versus Ca^2+^-normal environment. AFIF's action on PCSMS was most obvious in low Ca^2+^ environment, and the high concentration of Ca^2+^ weakened the diastolic effect of AFIF on PCSMS. When AFIF concentration reached 10000 *μ*g/mL, the PCSMS contraction amplitude in low Ca^2+^ environment was significantly lower than Ca^2+^-normal environment (*P* < 0.01), while the PCSMS contraction amplitude in high Ca^2+^ environment was significantly higher compared with the Ca^2+^-normal environment (*P* < 0.01).

**Figure 4:**
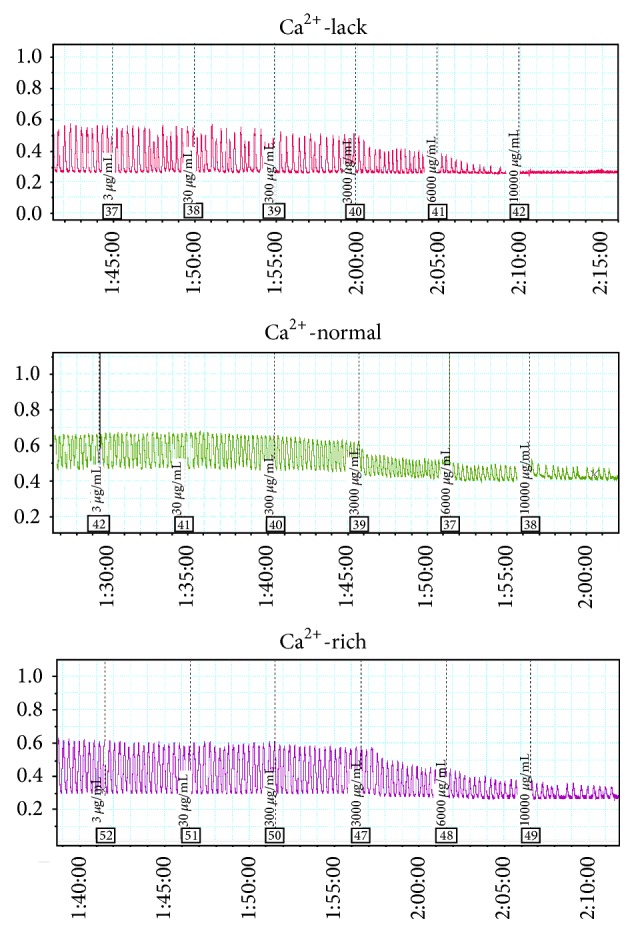
Effect of AFIF on spontaneous contraction of PCSMS in different Ca^2+^ environment recorded by PL 3508-0028 four-channel recorder. Low concentration (below 3000 *μ*g/mL) of AFIF had no influence on PCSMS contraction. When the concentration of AFIF reached 3000 *μ*g/mL, 6000 *μ*g/mL, and 10000 *μ*g/mL, AFIF decreased the mean contraction amplitude of PCSMS clearly. The diastolic effect of AFIF on PCSMS was most obvious in Ca^2+^-lack environment, and high Ca^2+^ concentration clearly weakened AFIF's action.

The mean contraction amplitude of PCSMS was decreased by AFIF treatment compared with control. This effect of AFIF was concentration-dependent, which became significant when the concentration of AFIF reached 3000 *μ*g/mL, 6000 *μ*g/mL, and 10000 *μ*g/mL (*P* < 0.05, *P* < 0.01) (Table 2 and Figure 4). This diastolic effect of AFIF on PCSMS was most obvious in Ca^2+^-lack environment, and high Ca^2+^ concentration significantly weakened AFIF's action (*P* < 0.01) (Figures 3 and 4).

### 4.3. AFIF (10000 *μ*g/mL) Showed an Inhibitory Action on the Mean Contractile Frequency of PCSMS

AFIF showed a concentration-dependent inhibitory action on the mean contractile frequency of PCSMS, but it is significant only when the concentration of AFIF reached 10000 *μ*g/mL (Table 3).

### 4.4. AFIF Corrected the Enhanced Contraction Amplitude of PCSMS Induced by Acetylcholine Chloride or Neostigmine and Further Reduced the Diminished Contraction Amplitude of PCSMS Induced by Atropine

Pretreatment of acetylcholine chloride (0.35 *μ*mol/L) and neostigmine (1 *μ*mol/L) increased the baseline of PCSMS contraction amplitude, while atropine (1 *μ*mol/L) decreased it. AFIF corrected the enhanced contraction of PCSMS induced by acetylcholine chloride or neostigmine and further reduced the diminished contraction amplitude of PCSMS induced by atropine (Table 4).

### 4.5. AFIF Showed No Significant Effect on the Mean Contractile Frequency of PCSMS after the Pretreatment of Acetylcholine Chloride, Neostigmine, or Atropine

AFIF showed no significant inhibitory effect on the mean contractile frequency of PCSMS after pretreatment (Table 5).

### 4.6. L-NAME Significantly Weakened AFIF's Action on the Contraction Amplitude of PCSMS

**Figure 5:**
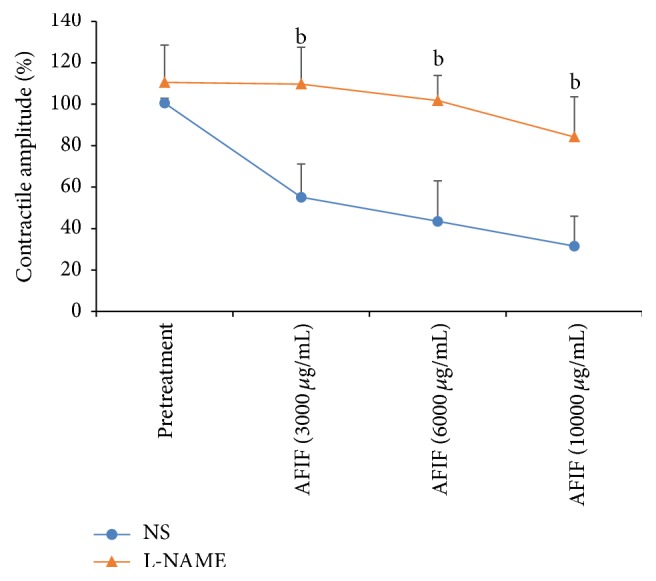
AFIF's action on PCSMS contraction amplitude after the pretreatment of L-NAME. *n* = 6, ^b^ *P* < 0.01 versus control. Neither L-NAME nor NS significantly changed the baseline of PCSMS contraction amplitude (*P* > 0.05). However, after the incubation of L-NAME for 20 min, the effect of AFIF on PCSMS contraction amplitude was inhibited. When AFIF concentration reached 3000 *μ*g/mL, 6000 *μ*g/mL, and 10000 *μ*g/mL, the mean contraction amplitude of PCSMS incubated with L-NAME was significantly higher than control (*P* < 0.01).

Comparing to the incubation of NS (control), L-NAME (10^−4^ mol/L, 20 min) significantly reduced the relaxation of PCSMS induced by AFIF (Figure 5).

### 4.7. AFIF Significantly Increased the cGMP and PKG Levels of PCSMS

Comparing with NS incubation, AFIF (6000 *μ*g/mL) incubation for 30 min significantly increased the cGMP and PKG levels of PCSMS measured by ELISA kits (*P* < 0.01, *P* < 0.05) (Table 6).

## 5. Discussion

Recently, Chinese herbals have been paid a close attention as effective treatments of functional dyspepsia [6], and some of them have been proved to regulate the contraction of gastrointestinal isolated muscle strips [10–12]. AFI, a member of the family Rutaceae, named as “Zhishi” in Chinese medicine, is used to “Po-Qi Chu-Pi (regulating qi-flowing and treating fullness)” and improve the symptoms of dyspepsia. It has been proved to promote gastric emptying process [13] and becomes a commonly used drug for the treatment of functional dyspepsia. It also has been verified by in vitro experiment that AFI decreases the contractility of isolated gastric muscle strips in rats [14]. AFIF, the major effective constituent of AFI [8], has been reported to promote gastric emptying in rats as well [9]. However, the mechanisms of AFIF's therapeutic effect have not been clarified, and AFIF's action on isolated gastric muscle strips has not been published yet.

Gastric emptying dysfunction is considered to be an important pathogenic factor in functional dyspepsia, which induces clinical symptoms of FD [5, 15]. However, existing prokinetic agents are not always satisfactory to promote the gastric emptying process of FD patients. Recent studies confirmed that some FD patients' gastric contractility is hyper rather than diminished [16], and the hyper pyloric contractility can cause the dysfunction of gastric emptying [17, 18]. Some experts argued that pylorus is an independent unit of resistance in gastric emptying process [19], and relaxing pyloric sphincter muscle is a feasible way to regulate gastrointestinal motility and improve FD symptoms [20].

As we know, the intracellular Ca^2+^ homeostasis is the basis for maintaining normal contraction and relaxation of smooth muscle. Extracellular Ca^2+^ can be charged or discharged by muscle cells through the cell membrane, leading to the increase or decrease of intracellular Ca^2+^ concentration, which causes the contraction or relaxation of smooth muscle. It is well known that nitric oxide (NO) is one of the most important regulatory factors in alimentary tract which adjust the gastrointestinal motility. It activates intracellular guanylate cyclase to catalyze guanosine triphosphate (GTP) and generate cGMP, which further activates PKG to prevent the influx of extracellular Ca^2+^ and induces a decrease of intracellular Ca^2+^ concentration, causing smooth muscle relaxation. The reported study also demonstrated that NOS decreases Ca^2+^ concentration in muscle cells by upregulating cGMP level [21].

Previous animal experiments have proved that AFIF upregulates NO concentration in serum [22] and NO can accelerate gastric emptying by inhibiting the contraction of pylorus [23]. According to a recent study [24],* Poncirus fructus*, another member of the family Rutaceae which is closely related to AFI, can reduce the smooth muscle contraction of experiment animals, and this action can be significantly inhibited by L-NAME.

In this study, AFIF did not show any significant effect on the contraction of ALSMS, but it showed a concentration-dependent inhibitory action on the spontaneous contraction of PCSMS. AFIF's inhibitory action on the mean contraction amplitude of PCSMS was significant when its concentration reached 3000 *μ*g/mL, 6000 *μ*g/mL, and 10000 *μ*g/mL. And its effect on the contractile frequency of PCSMS was significant only when its concentration reached 10000 *μ*g/mL. In another experiment, we used Ach and neostigmine to induce the enhancing of PCSMS contraction which can partly simulate the abnormally enhanced contraction of pylorus. In this experiment, AFIF effectively corrected the pathological condition of PCSMS contraction. Our research also showed that AFIF's action was negatively correlated with Ca^2+^ concentration in the bath, which demonstrated that the diastolic effect of AFIF on PCSMS is likely to be related to inhibiting extracellular Ca^2+^ influx and downregulating intracellular Ca^2+^ concentration. Our results also showed that L-NAME significantly weakened AFIF's diastolic effect on PCSMS contraction. And AFIF (6000 *μ*g/mL) incubation increased the levels of cGMP and PKG in PCSMS significantly. These results demonstrated that the diastolic effect of AFIF on PCSMS is probably achieved by activating NOS and associated with upregulating cGMP and PKG levels. However, the specific composition of AFIF working in this process still remains to be further studied.

## 6. Conclusions

As the major effective constituent of AFI, AFIF has a diastolic effect on PCSMS. This effect of AFIF is closely related to activating NOS, upregulating cGMP and PKG levels, and downregulating intracellular Ca^2+^ concentration of smooth muscle. Our study may partly elucidate the mechanism of AFIF's action in the treatment of FD and provide some ideas for the complementary and alternative therapies.

## Figures and Tables

**Table 1:** AFIF's action on the mean contractile frequency of ALSMS (mean ± SD).

Administration	*N*	Mean contractile frequency (beat per minute, BPM)
NS (control)	9	6.26 ± 0.60
AFIF, 3 *μ*g/mL	9	6.39 ± 0.97
AFIF, 30 *μ*g/mL	9	6.03 ± 0.49
AFIF, 300 *μ*g/mL	9	5.88 ± 0.75
AFIF, 3000 *μ*g/mL	9	5.96 ± 1.23
AFIF, 6000 *μ*g/mL	9	5.69 ± 0.81
AFIF, 10000 *μ*g/mL	9	5.46 ± 0.88

**Table 2:** AFIF concentration dependently reduced the mean contraction amplitude of PCSMS in different Ca^2+^ environment (mean ± SD).

Administration	*N*	Mean contraction amplitude (%)		
		Ca^2+^-lack	Ca^2+^-normal	Ca^2+^-rich
NS (control)	10	100.03 ± 3.40	101.81 ± 3.35	97.38 ± 7.35
AFIF, 3 *μ*g/mL	10	98.06 ± 4.97	97.60 ± 2.92	98.26 ± 6.47
AFIF, 30 *μ*g/mL	10	95.04 ± 11.34	95.39 ± 7.71	93.92 ± 5.16
AFIF, 300 *μ*g/mL	10	84.47 ± 19.37	82.88 ± 19.89	81.85 ± 10.32
AFIF, 3000 *μ*g/mL	10	59.96 ± 22.09^b^	52.60 ± 17.82^b^	70.95 ± 22.02^a^
AFIF, 6000 *μ*g/mL	10	34.15 ± 31.03^b^	40.58 ± 18.29^b^	47.45 ± 17.39^b^
AFIF, 10000 *μ*g/mL	10	3.96 ± 9.00^b^	24.31 ± 16.90^b^	34.78 ± 16.33^b^

^a^ *P* < 0.05 and ^b^ *P* < 0.01 versus control.

**Table 3:** AFIF's action on the mean contractile frequency of PCSMS (mean ± SD).

Administration	*N*	Mean contractile frequency (beat per minute, BPM)		
		Ca^2+^-lack	Ca^2+^-normal	Ca^2+^-rich
NS (control)	10	3.58 ± 0.79	3.57 ± 0.56	4.30 ± 0.83
AFIF, 3 *μ*g/mL	10	3.63 ± 0.74	3.52 ± 0.53	4.37 ± 0.96
AFIF, 30 *μ*g/mL	10	3.68 ± 0.90	3.49 ± 0.54	4.28 ± 0.81
AFIF, 300 *μ*g/mL	10	3.57 ± 0.60	3.39 ± 0.74	4.37 ± 0.91
AFIF, 3000 *μ*g/mL	10	3.62 ± 1.47	3.49 ± 1.09	4.69 ± 0.89
AFIF, 6000 *μ*g/mL	10	2.16 ± 2.10	3.0 ± 0.96	4.16 ± 0.74
AFIF, 10000 *μ*g/mL	10	0.62 ± 1.40^b^	1.63 ± 1.30^b^	2.75 ± 1.60^b^

^b^ *P* < 0.01 versus control.

**Table 4:** Effect of AFIF on mean contraction amplitude of PCSMS after pretreatments of Ach, neostigmine, or atropine (mean ± SD).

Administration	*N*	Mean contraction amplitude (%)		
		Acetylcholine chloride	Neostigmine	Atropine
Pretreatment	7	197.15 ± 35.45	501.95 ± 109.74	70.89 ± 11.04
AFIF, 3 *μ*g/mL	7	156.05 ± 17.67	422.00 ± 153.11	72.54 ± 13.81
AFIF, 30 *μ*g/mL	7	154.02 ± 24.67	418.91 ± 128.05	70.80 ± 14.47
AFIF, 300 *μ*g/mL	7	156.94 ± 28.02	384.70 ± 79.80	67.23 ± 17.47
AFIF, 3000 *μ*g/mL	7	130.39 ± 19.52^b^	299.94 ± 82.59^a^	45.08 ± 14.38^b^
AFIF, 6000 *μ*g/mL	7	90.40 ± 22.20^b^	166.69 ± 76.85^b^	38.80 ± 19.69^b^
AFIF, 10000 *μ*g/mL	7	52.64 ± 23.01^b^	76.62 ± 65.47^b^	15.03 ± 22.90^b^

^a^ *P* < 0.05 and ^b^ *P* < 0.01 versus pretreatment.

**Table 5:** Effect of AFIF on mean contractile frequency of PCSMS after pretreatments of Ach, neostigmine, or atropine (mean ± SD).

Administration	*N*	Mean contractile frequency (beat per minute, BPM)		
		Acetylcholine chloride	Neostigmine	Atropine
Pretreatment	7	4.36 ± 1.51	5.26 ± 1.48	4.11 ± 0.85
AFIF, 3 *μ*g/mL	7	4.43 ± 1.43	5.63 ± 2.24	4.11 ± 0.70
AFIF, 30 *μ*g/mL	7	4.25 ± 1.49	5.04 ± 0.78	4.15 ± 0.67
AFIF, 300 *μ*g/mL	7	4.23 ± 1.41	4.51 ± 0.60	4.18 ± 0.54
AFIF, 3000 *μ*g/mL	7	4.55 ± 1.35	4.19 ± 0.39	4.28 ± 0.77
AFIF, 6000 *μ*g/mL	7	4.24 ± 1.30	4.05 ± 0.88	3.48 ± 1.52
AFIF, 10000 *μ*g/mL	7	3.52 ± 2.05	3.36 ± 1.59	1.37 ± 1.76

**Table 6:** Effect of AFIF incubation on the cGMP and PKG levels of PCSMS (mean ± SD).

Incubation	*N*	cGMP (pg/mg)	PKG (nM/mg)
NS	7	1.51 ± 0.15	27.41 ± 3.53
AFIF (6000 *μ*g/mL)	7	1.78 ± 0.12^b^	31.60 ± 3.44^a^

^a^ *P* < 0.05 and ^b^ *P* < 0.01 versus control.
